# Enhancing Salt Stress Tolerance in Tomato (*Solanum lycopersicum* L.) through Silicon Application in Roots

**DOI:** 10.3390/plants13101415

**Published:** 2024-05-19

**Authors:** Borja Ferrández-Gómez, Juana D. Jordá, Mar Cerdán, Antonio Sánchez-Sánchez

**Affiliations:** 1 Department of Biochemistry and Molecular Biology, Edaphology and Agricultural Chemistry, University of Alicante, 03080 Alicante, Spain; borja.ferrandez@ua.es (B.F.-G.); juana.jorda@ua.es (J.D.J.); mar.cerdan@ua.es (M.C.); 2 Institute for Environmental Studies Ramon Margalef, University of Alicante, 03690 Alicante, Spain

**Keywords:** root, silicon, tomato, salt stress, hydroponic culture

## Abstract

Soil salinization poses a significant threat to agricultural productivity, necessitating innovative agronomic strategies to mitigate its impact. This study focuses on improving salt stress resistance in tomato plants through the application of silicon (Si) in roots. A greenhouse experiment was carried out under normal conditions (control, and 1 and 4 mM Si) and under salinity stress (salt control, and 1 and 4 mM Si). Various parameters were analyzed in leaves and roots. Under normal conditions, tomato plants grown in non-saline conditions exhibited some toxicity when exposed to Na_2_SiO_3_. As for the experiments under salt stress conditions, Si mitigated oxidative damage, preserving root cell membrane integrity. The concentration of malondialdehyde was reduced by 69.5%, that of proline was reduced by 56.4% and there was a 57.6% decrease in catalase activity for tomato plants treated with 1 mM Si under salt stress. Furthermore, Fe uptake and distribution, under salt conditions, increased from 91 to 123 mg kg^−1^, the same concentration as that obtained for the normal control. In all cases, the lower dose produced better results under normal conditions than the 4 mM dose. In summary, this research provides a potential application of Si in non-fertigated crop systems through a radicular pathway.

## 1. Introduction

Due to changes in climate conditions (e.g., damage caused by different biotic and abiotic stresses and population increase) and due to new regulations (Regulation (EU) 2019/1009 of the European Union [1]), agronomic yield and productivity are facing a delicate situation that requires balancing to ensure their sustainability. To achieve this goal, scientists and companies are seeking solutions through the use of agrochemical products to reduce the effects of stress on fruits and vegetables.

Silicon (Si) is the second most abundant element in the Earth’s crust and is present in the form of silicates, oxides and orthosilicic acid. However, its availability in soil varies greatly due to its slow solution from minerals, soil adsorption and intensive agricultural practices, which significantly reduce the available levels of this mineral. One of the most relevant forms is orthosilicic acid (H_4_SiO_4_), the concentration of which in soil solution ranges from 0.09 to 23.4 mg L^−1^ [2].

Silicon is considered a non-essential element for plant nutrition [3], and Si-based biostimulants are showing optimal results in terms of the plant growth and development of many species [4,5,6,7,8]. Numerous studies have shown that Si increases plant tolerance to various types of biotic [9] and abiotic stresses that cause a decrease in the average yield and production of major crops by more than 50% each year [10]. Specifically, its effectiveness has been highlighted in situations of stress caused by water stress [11], temperature [10], nutrient imbalance [12], and heavy metal toxicity [13]. However, one of the most severe impacts on crops worldwide is caused by salt stress due to its high impact, significantly damaging plants, limiting their growth, productivity and crop quality worldwide [11,14,15]. Si in foliar and/or radicular applications has shown high effectiveness in reducing salinity problems in crops such as barley [16], wheat [17], maize [11], sugarcane [18], alfalfa [19], mango [20], cucumber [21] and tomato [5,22,23,24].

The mechanisms that take place in reducing salt stress are explained by the limitations to NaCl uptake due to Si action, maintaining stomatal structure, improving photosynthetic activity, reducing free proline content, and modifying antioxidant enzyme production [25]. Additionally, stress conditions are alleviated through reduced lipid peroxidation in plants via increased antioxidant enzymes and non-enzymatic antioxidant activity [5,16,26] and increased root activity, which improves nutritional balance [6].

One of the most widely grown and economically valuable vegetables worldwide is tomato (*Solanum lycopersicum* L.). Considered a moderately salt-tolerant species, it experiences a significant decline in growth and yield under high salinity levels as it is glycophytic [27]. To counteract this effect, it has been demonstrated that Si fertilization in the form of diatomite [10,28], Na_2_SiO_3_ [10,23,24,29,30], nano-silicon [22,29], Na_2_Si_3_O_7_ [31], Na_2_SiO_3_·9H_2_O [32] and H_4_SiO_4_ [33] has shown positive responses associated with increased production, increased post-harvest quality, and a decrease in parameters related to salt stress. Silicon has also been reported to improve tomato growth and yield in soilless cultivation systems, the most widely used form of tomato production in southern Europe. However, the beneficial effects of Si on plants subjected to salt stress in these systems have shown contradictory results [4,27,33,34]. Thus, further studies are required.

This study aims to expand the knowledge of the effect of Na_2_SiO_3_ on tomato plants under salt stress in hydroponic culture. Specifically, the objectives of this study were as follows: (i) to study whether the root application of Si has any effect on non-stressed seedlings, (ii) to evaluate the effectiveness of Si in combating the effects caused by salinity on seedlings, and (iii) to determine the most appropriate concentration of Si to be applied in hydroponics to counteract salt stress.

## 2. Results

Table 1 shows the ANOVA *p*-values for the two fixed factors (Si concentration (D) and salinity (S)), as well as the (D) × (S) interaction. As can be seen, there were different parameters evaluated in this work that were affected by the two factors or their interaction, indicating that some parameters did not show significant differences throughout the experiment.

In particular, growth parameters such as fresh weight (FW), dry weight (DW) and plant length (PL), as well as Na and Mn leaf concentration, did not show significant differences from the interaction of the two factors. Therefore, these parameters were analyzed individually for the dose of Si applied (0, 1 and 4 mM) and the type of growth conditions (non-saline and saline).

It is important to highlight that significant differences can be observed for the interaction of both factors for the rest of the macro- (Ca, K and Mg) and micronutrients (Fe, Cu and Zn), as well as for chlorophyll content, parameters used to evaluate oxidative stress (%EC_1_/EC_2_ and malondialdehyde), osmotolerant parameters (proline and total soluble sugars) and catalase activity.

### 2.1. Effects of Si on Plant Growth, Micro- and Macronutrients and Chlorophyll Parameters in Plant Development under Salt Stress

Plant growth was significantly reduced by the salt stress treatment with 50 mM NaCl, as evidenced by the FW, DW and PL (Table 2). Regarding the effect of Si applied to the root, FW and PL parameters showed a similar trend with a slight improvement, with the 1 mM Si dose obtaining the best results (Table 2). The application of Si did not affect the Na foliar concentration, while the Mn concentration was slightly reduced, although no deficiency symptoms were observed in the plant.

Regarding the influence of salinity, Table 2 shows that plants grown in this medium had higher Na and lower Mn concentrations.

The mineral concentration of macronutrients (Ca, K and Mg) in the aerial part of tomato plants was significantly modified by salinity (Table 3). In seedlings grown under salt stress, the application of Si slightly but significantly increased the concentration of Ca for the highest Si dose. For K and Mg, the 1 mM dose significantly increased their concentration compared with the salt control, and K levels were similar to those of non-saline condition.

In tomato seedlings grown under non-saline conditions, the addition of Si had no negative effect on the concentration of micronutrients (Fe, Cu and Zn) as can be seen in Table 3. On the other hand, plants subjected to salt stress significantly reduced the values of all these elements. The application of radicular Si as an alleviator of this stress significantly improved the Fe concentration until it reached control values.

Chlorophyll was greatly reduced under salt stress (Table 3), although Si partially avoided this reduction. It is worth noting that no significant differences were observed between the two doses. Additionally, no significant effect of Si was observed under non-stress conditions at either dose compared with the control.

### 2.2. Effects of Si on Oxidative Stress in Plant Development under Salt Stress

**Figure 1:**
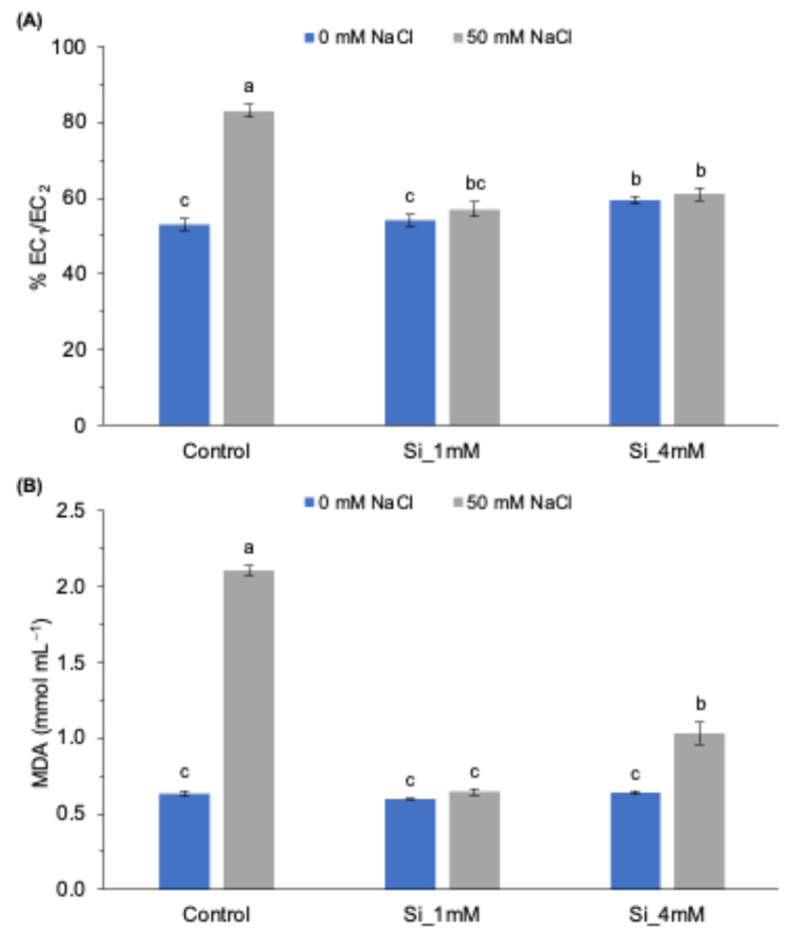
(**A**) Effect of Si (Na_2_SiO_3_) concentration on membrane permeability and (**B**) MDA concentration in tomato plants grown under normal and salt stress conditions. Means followed by the same letter are not significantly different according to Tukey’s multiple comparison test (*p* < 0.05). The bars show the standard deviation of the mean (*n* = 4).

The cell membrane is damaged by the excessive accumulation of Na^+^ and Cl^−^ ions in plant tissues when grown under salt stress. Additionally, the loss of electrolytes is directly related to the plasmatic membrane permeability of the cells, so the more damaged the membrane is, the greater the loss of electrolytes (Figure 1A). Specifically, the %EC_1_/EC_2_ in salt control was 30% higher than that in the normal control. The addition of Si had a positive effect on the integrity of these membranes in plants grown under salt solutions; silicon significantly reduced the rate of electrolyte leakage by 31.3% and 26.6% for Si_1mM and Si_4mM, respectively. Si did not cause any effect on the membrane integrity of non-saline tomato plants at the lower dose, but a slight increase was achieved for the higher dose.

Another parameter used to evaluate oxidative damage is malondialdehyde (MDA). From Figure 1B, it can be observed that non-stressed plants had the lowest concentrations of MDA, and there were no significant differences among the three treatments, indicating that the application of Si under these conditions did not have negative effects due to phytotoxicity. However, compared with the increasing salt stress levels in plants, the concentration of MDA was over three times higher in the salt control. However, Si application reduced the levels of MDA in plants under salt stress, both at the 1 and 4 mM doses. This effect was remarkably within the lower dose.

### 2.3. Effects of Si on Osmotolerance in Plant Development under Salt Stress

**Figure 2:**
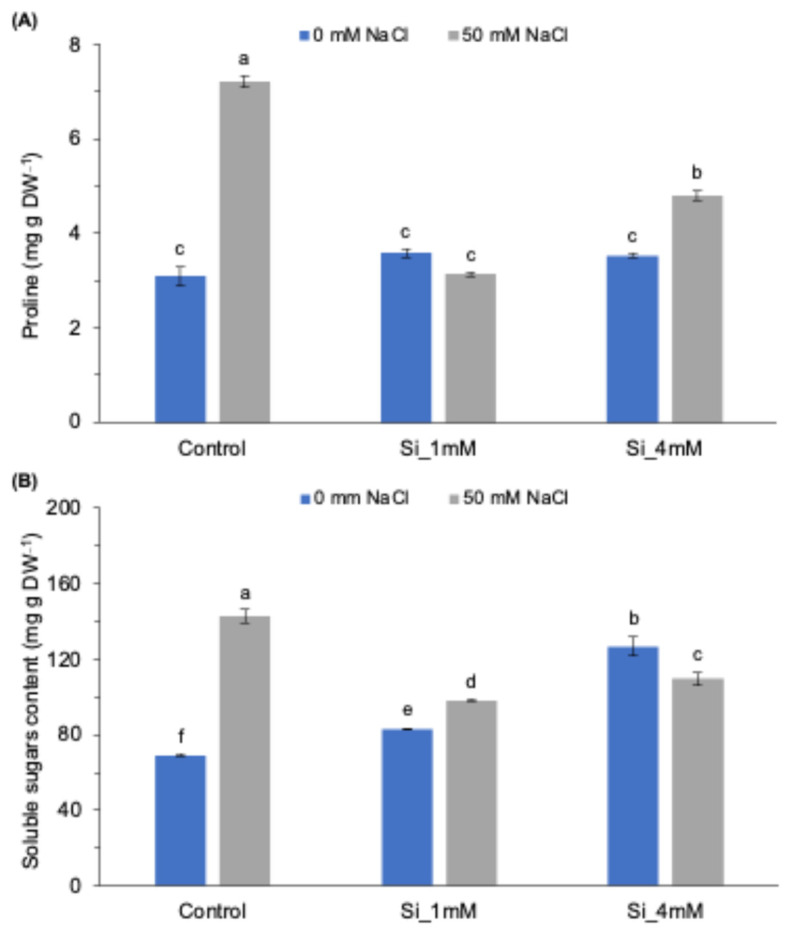
(**A**) Effect of Si (Na_2_SiO_3_) concentration on proline content and (**B**) soluble sugars content in tomato plants grown under normal and salt stress conditions. Means followed by the same letter are not significantly different according to Tukey’s multiple comparison test (*p* < 0.05). The bars show the standard deviation of the mean (*n* = 4).

To evaluate the effect of Si application on the root, two important parameters for plant osmotolerance, proline and total soluble sugars were also determined. Figure 2A shows the variation in proline concentration with respect to the Si dose for plants grown under normal and salt stress conditions.

The results show that the proline content in the normal treatments, regardless of Si application, was quite similar. However, the amount of this amino acid increased by more than 2.5 times in the stress control plants. The addition of Si to the saline medium had a remarkably positive effect, since proline accumulation was significantly reduced in these treatments, even to the point that there were no significant differences compared with the control treatment when the solution was supplied with both concentrations. This behavior agrees with the MDA concentration obtained, suggesting that these plants withstood a lower level of stress.

As for soluble sugars, it is important to note that they are osmotically active compounds synthesized and accumulated by plants to counteract the effects of high ion concentrations and facilitate water uptake. As shown in Figure 2B, the content of foliar sugars was significantly higher in plants under salt stress than in those grown in normal conditions. Thus, the high salt concentration in the growing medium contributed to a rise in the sugar content. Under normal conditions, the application of Si did not seem to be beneficial to the plant, although it had no remarkable effects either, since there was a slight increase in sugar content. However, when plants were stressed by salinity, there was a significant increase in the amount of sugars in the leaves. In these conditions, Si did show a beneficial effect, since the levels were reduced to values close to those of the control treatment, although significantly different. It should be noted that this effect, again, was greater when applied at a 1 mM dose, since the decrease in sugar content was higher with respect to the 4 mM dose of Si.

### 2.4. Effects of Si on Enzymatic Activity in Plant Development under Salt Stress

**Figure 3:**
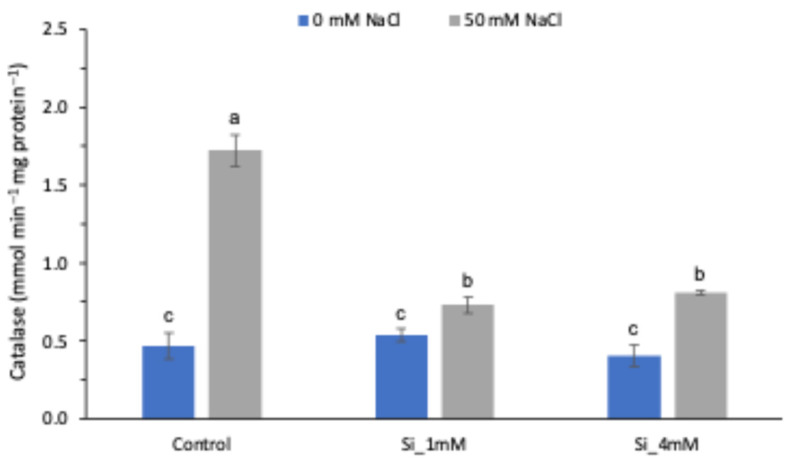
Effect of Si (Na_2_SiO_3_) on CAT activity in tomato plants under normal and salt stress conditions. Means followed by the same letter are not significantly different according to Tukey’s multiple comparison test (*p* < 0.05). The bars show the standard deviation of the mean (*n* = 4).

As can be seen in Figure 3, catalase (CAT) activity increased in the leaves of tomato plants under salt stress. The addition of Si to these plants reduced enzyme activity by 57.6% and 53.1% for the 1 and 4 mM doses, respectively, and in all cases, the difference was statistically significant. The decrease in CAT activity was considerable, reaching levels similar to those of normal treatments. This finding is consistent with the variations in the parameters studied in this work, where the 1 mM dose of Na_2_SiO_3_ was demonstrated to be more effective than the 4 mM dose. Finally, it should be noted that Si did not affect the enzymatic activity of the plants grown under normal conditions.

## 3. Discussion

The results of this work showed that the root application of silicon, Na_2_SiO_3_, to tomato plants grown under salt stress conditions had a positive effect on plant growth, as can be seen for FW and PL in Table 2. In particular, compared with the salt control, it produced a decrease in the levels of membrane permeability, MDA, proline, total soluble sugars and the enzyme activity of CAT, especially when applied at the lowest dose (Figure 1, Figure 2 and Figure 3).

It should be noted that, under normal conditions, Si application did not bring about a significant improvement in the plant growth, oxidative, osmotolerant and enzyme activity parameters in tomato plants. This suggests that the Na^+^ content provided by the silicon salt may have produced minor salinity stress in normal plants and inhibited their enhanced development. The reduction in Na^+^ uptake and the maintenance of the optimal Na^+^/K^+^ ratio are considered to be the most significant processes responsible for the salt tolerance mediated by Si [35]. In this work, Na^+^/K^+^ ratios of 0.037, 0.048 and 0.065 (control, and 1 and 4 mM respectively) were obtained for plants grown under normal conditions, and 2.00, 1.14 and 1.62 (salt control, 1 and 4 mM, respectively) ratios were obtained for plants under salinity. These differences could explain the behavior of exogenous Si applied at the root level in normal plants and its effectiveness in tomato plants under salt stress.

This is reinforced by the fact that the higher dose (4 mM) did cause a slight increase in the values of membrane permeability (Figure 1A) and CAT enzyme activity (Figure 3). This behavior of Si under normal conditions was discussed in the review by Cheraghi et al. [36], where it was noted that the beneficial effects of Si are mainly obtained under salt and drought stress conditions and not so much in plants without abiotic stress.

In our study, we observed a decrease in growth, weight and chlorophyll content in salt plants compared with normal plants (Table 2 and Table 3). However, the addition of 1 mM Si allowed the recovery of these values to better ones than those observed for the higher dose. This trend was the same as that observed by Haghighi et al. [22], where 1 mM Si application caused a significant increase in tomato plant growth and chlorophyll content due to the lower toxicity of Na^+^ and Cl^−^ in leaves, which is correlated with stomatal enclosure as a limiting factor of the amount of photoassimilate production and efficiency of photosystem II [6,22,24,37].

On the one hand, increased K^+^ uptake and transport and decreased Na^+^ absorption and transport from roots to aerial parts in barley were found to be due to the Si-induced stimulation of plasma membrane H^+^-ATPase under salt stress [5,26,38]. On the other hand, different authors suggested that soluble Si can be absorbed by roots and act as a secondary pathway to modulate defense responses to salt stress [29,39,40,41]; for this reason, better results have been obtained in the application of Si in plants grown under stress.

Furthermore, Gou et al. [32] concluded that Si addition can decrease salt stress-induced chlorophyll degradation in tomato, delaying leaf senescence in these plants, due to increased cytokinin concentrations.

Regarding macro- and micronutrient content, the increase in Fe concentration in stressed and Si-treated plants is noteworthy, reaching the level of Fe in normal control plants. This impact on Fe uptake is due to the fact that Si is a nutrient that benefits its adsorption and distribution due to its high surface area [42]. Once again, the 1 mM dose showed better results than the 4 mM dose due to the lower amount of Na^+^ present in the plant as overaccumulation of Na limits the uptake of other nutrients [6].

As for the reduction in membrane permeability and MDA values, the decrease shown in Figure 1B occurred because Si application at the root level induced less lipid peroxidation and thus maintained higher plasma membrane integrity (Figure 1A). This was previously reported in Si-treated barley and tomato plants, as they showed a decrease in MDA accumulation under heat stress conditions [10,30] and in salt-stressed rice crops [6].

Another target molecule in the response to salt stress is proline, as it has high antioxidant activity and is a scavenger of reactive oxygen species (ROS) [4,43], thereby preventing oxidative damage to membranes, as can be seen in Figure 1A and Figure 2A. Consequently, it is important to note the decrease in proline accumulation in the Si-treated stressed plants, recovering back to the values obtained in the normal control for the 1 mM dose (Figure 2A). This is a frequent response of plants and is attributed to a reduction in stress levels and salinity toxicity due to this osmoregulatory molecule [31,44]. This trend was the same as that observed for the concentration of soluble sugars, where there was an increase in the salt control and, after Si application, a significant reduction, although it did not reach the concentration of the normal control (Figure 2B). This behavior has been studied previously and is again associated with the role of tomato in accumulating sugars and scavenging ROS [4,45].

Furthermore, as can be observed in Figure 3, the concentration of CAT activity was significantly lower in Si-treated stressed plants compared with control plants as a result of improved membrane integrity due to Si modulation, as suggested by different authors for experiments carried out on tomato, rice, spinach and cucumber [6,24,29].

On the other hand, it has been shown that the application of Si from marine extracts on tomato and cucumber seeds significantly alleviated the effects of salt stress, improving various parameters such as tomato growth, photosynthetic pigment and soluble protein content, the photosynthetic rate and root morphological traits [6,46], reinforcing the results obtained in this work indicating the advantages of root application over the foliar application of this type of fertilizer in tomato crops.

## 4. Materials and Methods

### 4.1. Plant Material, Cultural Conditions and Treatment

Seedlings of tomato (cv Seny F1) were used in this research. Plant development was carried out in the greenhouse facilities of the University of Alicante (38°23′05″ N–0°30′47″ W) under controlled conditions of temperature and relative humidity (18 °C/25 °C (night/day) and 70% RH). In addition, the greenhouse facilities were equipped with an independent climate control system (air conditioning, heating, a humidifier, and an evaporator) and assimilation lights.

Seed germination was carried out on silica sand as an inert substrate, sterilized and previously moistened with a 1 mM CaSO_4_ (Merck KGaA, Darmstadt, Germany) solution to prevent the development of fungi. This phase was carried out under controlled conditions (18 °C/25 °C (night/day) and 70% RH) in a plant growth chamber (Sanyo MLR-350, Tokyo, Japan) with irrigation using distilled water every 2 days. After two weeks, 54 seedlings were transplanted into the hydroponic experimental unit. Each seedling was placed in a 250 mL polyethylene vessel through a hole made on the cover. A pure hydroponic system was used. The composition of the nutrient solution was as follows: 3.5 mM Ca(NO_3_)_2_, 1.25 mM MgSO_4_, 4.5 mM KNO_3_, 0.75 mM K_2_SO_4_, 1.5 mM KH_2_PO_4_, 0.5 mM NH_4_NO_3_, 0.31 µM CuSO_4_ 5H_2_O, 1.36 µM ZnSO_4_, 12.7 µM MnSO_4_, 0.06 µM (NH_4_)_6_Mo_7_O_24_, 46.3 µM H_3_BO_3_ and Fe which was added in the form of FeEDDHA, 35.8 µM), in accordance with Cerdán et al. [47]. The pH of the nutrient solution was adjusted to 6.0 using KOH (Merck KGaA, Germany) or H_2_SO_4_ (VWR International Eurolab S.L., Barcelona, Spain).

Silicon was applied in the form Na_2_SiO_3_ (Merck KGaA, Germany) to plants grown under normal conditions (NC) and under salt stress conditions (SC) with 0 and 50 mM NaCl (VWR International Eurolab S.L., Spain), respectively. Three different Si solutions were applied to the roots to control salt effects on tomato growth (0, 1 and 4 mM), as can be seen in Table 4. Nine plants were used in each treatment.

In all cases, the roots were immersed in 250 mL of the nutrient solution described above, which was replaced by a new one when the electrical conductivity was ≤0.8 dS m^−1^. It should be noted that, for the desired treatments, Si and NaCl were added together with the nutrient solution.

### 4.2. Determination of Plant Growth, Macro- and Micronutrient Concentrations and Chlorophyll Content

After 30 days, the tomato plants were harvested, and the aerial part of the plants was rinsed several times with distilled water. Excess water was removed with paper, and plants were weighed to measure their fresh weight (FW) and plant length (PL). After that, samples were dried overnight at 60 °C in an oven and weighed (DW) again to obtain the dry weight.

Macro- and micronutrient content in plants was determined vua the calcination of 0.5 g of dry leaves at 550 °C and subsequent digestion with HCl 6 M (Merck KGaA, Germany). Digested ash was dissolved in osmotized water, resulting in a volume of 25 mL. These solutions were measured using an inductively coupled plasma optical emission spectrometer (ICP-OES, model 720-ES, Agilent Technologies, Santa Clara, CA, USA).

Chlorophyll content (total, a- and b-) in fresh leaves of tomato plants was measured using the method of Abadía et al. [48]: 1 g of fresh plant material with 0.1 g CaCO_3_ (Merck KGaA, Germany) and 25 mL of methanol (Merck KGaA, Germany) was left to stand for 4 h. The absorbance of the alcoholic extract was measured at 663 and 645 using a UV/VIS/NIR spectrophotometer (JASCO V-630, Jasco Analitica Spain, S.L., Madrid, Spain).

### 4.3. Membrane Permeability and Malondialdehyde Analysis

Membrane permeability was estimated based on electrolyte loss at the cellular level; leaves were cut into segments of 1 cm in length, and 0.3 g of material was placed in Falcon tubes with 30 mL of distilled water, shaken vigorously for 1 min and incubated in a thermostatic bath at 30 °C for 2 h. After that, electrical conductivity (EC_1_) was measured. The samples were then incubated for 15 min at 100 °C, and electric conductivity (EC_2_) was measured again. Electrolyte loss was calculated as the ratio of both electrical conductivities and expressed as a percentage.

The concentration of MDA in plants is an indicator of the oxidative degradation of lipids, so its determination provides information on the oxidative damage caused by salt stress to tomato seedlings. MDA concentration was determined following the method described by Shu et al. [49]: 0.3 g of leaves was homogenized in 3 mL of trichloroacetic acid (0.1% *v*/*v*, Merck KGaA, Germany). Subsequently, the sample was filtered, and 1 mL was taken and added to a 3·mL mixture in a reaction tube. The mixture consisted of trichloroacetic acid (10% *w*/*v*, Merck KGaA, Germany) and thiobarbituric acid (0.65% *w*/*v*, Merck KGaA, Germany) in the same proportions. The reaction tubes were then kept in a thermostatic bath at 95 °C for 25 min. After that, the samples were immersed in an ice bath for 10 min and centrifuged for 25 min at 10,000 rpm. Supernatant absorbance was measured via UV-Vis spectrophotometry (JASCO V-630, Jasco Analitica Spain, S.L.) at 532 and 600 nm.

### 4.4. Osmotolerant Parameters Analysis

Plants under salt stress accumulate proline and soluble sugars. Proline content in plants was analyzed using the colorimetric procedure described by Magné and Larher [50]. Lyophilized leaves (0.1 g) were homogenized with distilled water at 60 °C and centrifuged for 15 min at 10,000 rpm, at 4 °C. The supernatant (0.5 mL) was collected, and 2 mL of acidified ninhydrin (Merck KGaA, Germany) was added. The samples were incubated for 30 min at 100 °C followed by rapid cooling in an ice bath. Finally, 5 mL of toluene (Merck KGaA, Germany) was added to the samples, which were shaken and stored in the dark at room temperature for 4 h. UV-Vis absorbance (JASCO V-630, Jasco Analitica Spain, S.L.) was measured at 520 nm. Quantification was performed using a calibration curve, with proline (Merck KGaA, Germany) solutions expressing the final results in mg of proline per g of dry weight^−1^.

Finally, the soluble sugar content in tomato plants was measured in the leaves using the anthrone method. 0.5 g of dried leaf samples were homogenized in 50 mL of ethanol. Then, 100 µL of the extract was mixed with 3 mL of 0.15% anthrone solution and incubated in a water bath at 95 °C for 15 min. Finally, the absorbance of the samples was measured with a spectrophotometer (JASCO V-630, Jasco Analitica Spain, S.L.) at 625 nm, and the total sugar concentration of the samples was calculated using a glucose standard.

### 4.5. Enzymatic Activity

The assay on CAT enzymatic activity was carried based on the foliar extract method: 5 mL of MES-KOH (pH 6.0, Merck KGaA, Germany) buffer solution was added to 0.5 g of frozen leaves and left to stand for 2 h. Subsequently, the samples were centrifuged at 10,000 rpm for 10 min at 4 °C, and 1.5 mL of the extract was taken to measure CAT activity through the rate of decomposition of H_2_O_2_. For this purpose, the H_2_O_2_ concentration was determined using a UV-Vis spectrophotometer (JASCO V-630, Jasco Analitica Spain, S.L.) at 240 nm after 3 min of reaction.

### 4.6. Statistical Analysis

The results obtained were statistically evaluated via a factorial analysis of variance (two-way ANOVA) with IBM^®^ SPSS^®^ software (28.0 version, IBM, Armonk, NY, USA) using the salinity (0 or 50 mM NaCl) (S) and silicon concentration (0, 1 and 4 mM Na_2_SiO_3_) (D) as fixed factors. In this analysis, both the effect of each factor on the parameters analyzed and the interaction between factors were analyzed. Statistically different groups were tested using Tukey’s test (*p* < 0.05).

## 5. Conclusions

This research presents a promising strategy for mitigating the impact of soil salinization on tomato cultivation within a nutrient solution hydroponics system. Specifically, enhanced stress tolerance has been achieved through the root application of silicon. The findings highlight the importance of optimizing the silicon dose to achieve the desired effects. According to the results obtained, the presence of silicon in the nutrient solution at a concentration of 1 mM, the lowest of the doses proposed in this research, induced several adaptative responses in tomato seedlings to alleviate salinity stress. These responses include, reducing oxidative damage by decreasing reactive oxygen species, preserving the integrity of the root cell membrane and improving the absorption and distribution of Fe under salt stress conditions. Thus, this study provides a potential application of Si, emphasizing its significance in addressing the challenges posed by salinization in agriculture.

## Figures and Tables

**Table 1:** ANOVA *p*-values for the two fixed factors and their interaction for the different parameters studied in this work. *p* < 0.05 is not significant, and fixed factors are (D) Si concentration and (S): NaCl concentration.

Factor	(D)	(S)	(D) × (S)
FW (g)	<0.001	<0.001	0.287
DW (g)	0.189	<0.001	0.298
PL (cm)	<0.001	<0.001	0.065
Ca (%)	<0.001	<0.001	<0.001
K (%)	<0.001	<0.001	<0.001
Mg (%)	<0.001	<0.001	<0.001
Na (%)	0.396	<0.001	0.494
Fe (mg kg^−1^)	0.720	0.608	0.002
Cu (mg kg^−1^)	<0.001	<0.001	<0.001
Mn (mg kg^−1^)	<0.001	<0.001	0.175
Zn (mg kg^−1^)	0.286	<0.001	0.007
Chl total (mg g^−1^)	0.383	<0.001	0.003
Chl a (mg g^−1^)	0.211	<0.001	0.008
Chl b (mg g^−1^)	0.217	<0.001	0.008
EC_1_/EC_2_ (%)	<0.001	<0.001	<0.001
MDA (mmol mL^−1^)	<0.001	<0.001	<0.001
Proline (mg g DW^−1^)	<0.001	<0.001	<0.001
SSC (mg g DW^−1^)	<0.001	<0.001	<0.001
Catalase activity (mmol min^−1^ mg protein^−1^)	<0.001	<0.001	<0.001

**Table 2:** Effect of salt stress (0 and 50 mM NaCl) and root application of Si (0, 1 and 4 mM Na_2_SiO_3_) on parameters not statistically different in the interaction (D) × (S). Data, expressed in means ± SE (*n* = 9), followed by the same letter are not significantly different according to Tukey’s multiple comparison test (*p* < 0.05). Sig. ^1^ ns: sig > 0.05; ***: sig < 0.001.

Parameter	FW (g)	DW (g)	PL (cm)	Na (%)	Mn (mg kg^−1^)
NaCl (mM)					
0	31.8 ± 0.5 a	2.92 ± 0.06 a	29.1 ± 0.4 a	2.3 ± 1.9 b	107.8 ± 1.7 a
50	9.6 ± 0.5 b	0.72 ± 0.06 b	18.3 ± 0.4 b	52.6 ± 1.9 a	40.7 ± 1.7 b
Sig. ^1^	***	***	***	***	***
Na_2_SiO_3_ (mM)					
0	18.7 ± 0.5 b	1.73 ± 0.07	20.0 ± 0.5 b	25 ± 2	84 ± 2 a
1	22.8 ± 0.5 a	1.81 ± 0.07	25.9 ± 0.5 a	29 ± 2	70 ± 2 b
4	20.5 ± 0.5 b	1.93 ± 0.07	25.3 ± 0.5 a	29 ± 2	68 ± 2 b
Sig. ^1^	***	ns	***	ns	***

**Table 3:** Effect of salt stress (0 and 50 mM NaCl) and root application of Si (0, 1 and 4 mM Na_2_SiO_3_) on nutritional and chlorophyll parameters of hydroponically grown tomato plants. Data, expressed as means ± SD (*n* = 9), followed by the same letter are not significantly different according to Tukey’s multiple comparison test (*p* < 0.05). Sig ^1^ ns: sig > 0.05; **: 0.001 < sig < 0.01; ***: sig < 0.001.

Treatment	0 mM NaCl			50 mM NaCl			Sig. ^1^
	Control	Si_1mM	Si_4mM	Salt Control	Si_1mM	Si_4mM	
Ca (%)	30.9 ± 0.5 a	30.8 ± 0.9 a	22.6 ± 0.9 b	13.3 ± 0.5 d	13.8 ± 1.1 cd	15.4 ± 0.1 c	***
K (%)	51.0 ± 1.3 a	47.2 ± 1.9 a	39.7 ± 1.7 b	23.9 ± 0.1 c	49 ± 4 a	34 ± 3 b	***
Mg (%)	5.0 ± 0.1 c	5.3 ± 0.2 b	6.3 ± 0.1 a	2.2 ± 0.1 f	2.9 ± 0.1 d	2.5 ± 0.1 e	***
Fe (mg kg^−1^)	121 ± 3 a	104 ± 2 ab	94 ± 4 b	91 ± 4 b	123 ± 6 ab	114 ± 4 ab	**
Cu (mg kg^−1^)	6.0 ± 0.1 b	5.8 ± 0.2 b	6.7 ± 0.1 a	3.6 ± 0.1 d	4.9 ± 0.1 c	3.9 ± 0.4 d	***
Zn (mg kg^−1^)	29 ± 1 ab	27.5 ± 0.4 b	29.8 ± 1.3 a	22.4 ± 0.9 c	26.3 ± 1.0 b	23.3 ± 0.6 c	**
Chl total (mg g^−1^)	1.9 ± 0.2 a	1.7 ± 0.1 a	1.7 ± 0.1 a	0.6 ± 0.1 c	0.9 ± 0.1 b	0.9 ± 0.1 b	**
Chl a (mg g^−1^)	1.1 ± 0.2 a	1.0 ± 0.1 a	0.9 ± 0.1 a	0.3 ± 0.1 c	0.6 ± 0.1 b	0.5 ± 0.1 b	**
Chl b (mg g^−1^)	0.8 ± 0.1 a	0.7 ± 0.1 a	0.7 ± 0.1 a	0.3 ± 0.1 c	0.5 ± 0.1 b	0.6 ± 0.1 b	**

**Table 4:** Treatment tested for Si effect on salt- and non-stressed tomato plants.

Treatment	NaCl (mM)	Na_2_SiO_3_ (mM)
Normal control	0	0
Si_1mM	0	1
Si_4mM	0	4
Salt control	50	0
Si_1mM	50	1
Si_4mM	50	4

## Data Availability

The original contributions presented in the study are included in the article; further inquiries can be directed to the corresponding author.
